# Efficacy of *Curcuma longa* in treatment of postprandial distress syndrome: An open-label randomized-controlled trial

**DOI:** 10.12688/f1000research.20662.1

**Published:** 2019-10-30

**Authors:** Nicharat Sawangroj, Jiratha Budkaew, Bandit Chumworathayi

**Affiliations:** 1 Department of Social Medicine, Khon Kaen Hospital, Khon Kaen, 40000, Thailand; 2 Office of Clinical Epidemiology, Faculty of Medicine, Khon Kaen University, Khon Kaen, 40002, Thailand

**Keywords:** Functional dyspepsia, postprandial distress syndrome, global overall symptom scale, Curcuma longa, simethicone

## Abstract

**Background:** Proton pump inhibitors are effective for functional dyspepsia but ineffective in relieving postprandial distress syndrome. *Curcuma longa* might be effective for postprandial distress syndrome. The objective of this study was to compare the efficacy of *Curcuma longa* and simethicone for postprandial distress syndrome in an open-label randomized-controlled trial.

**Methods:** This trial was conducted between July 2018 and February 2019. In total, 78 patients were randomly assigned to receive 4 weeks of treatment with 750 or 1,500 mg oral *Curcuma longa* per day or 240 mg simethicone per day. The patients assessed their symptoms using the dyspepsia Global Overall Symptom scale at baseline, week 2, and week 4. After stopping medication for 2 weeks, the patients assessed recurrent symptoms and day of recurrence by themselves at the end of week 6.

**Results:** In total, 78 patients underwent randomization (27 in 750 mg *Curcuma longa*, 26 in 1500 mg *Curcuma longa*, and 25 in simethicone groups). After 2 weeks, there were no significant differences in all mean changes of symptoms scores (95%CI) of postprandial distress syndrome [-4.1 (-4.5, -2.6) vs -4.3 (-5.2, -3.3) vs -4.2 (-4.8, -3.5), P=0.954]. Over a period of 4 weeks, the reduction in mean scores was greater among participants receiving simethicone (although not statistically significant) compared with two intervention groups [-4.6 (-5.7, -3.6) vs -5.4 (-6.6, -4.1) vs -6.2 (-7.2, -5.2), P=0.122]. The rate of recurrence was significantly lower in simethicone than the two *Curcuma longa* groups (42.9 vs 45.5 vs 13.6%, P=0.047). There was no serious adverse event reported in all three groups.

**Conclusions:** *Curcuma longa* had a similar effect on treatment outcomes to simethicone after 2 and 4 weeks, but the recurrence rate of symptoms was significantly higher without serious adverse events.

**Registration:** Registered with the Thai Clinical Trials Registry on 31 January 2018; TCTR20180131001.

## Introduction

Dyspepsia is a common functional gastrointestinal disorder which affects 20% of the global population ^1^. Although dyspepsia is not a life-threatening condition, it disrupts the quality of life and also socioeconomic impaction for suffering patients ^2– 4^. The United States population spends $18 billion annually on dyspepsia management ^4^. In Thailand, the prevalence of dyspepsia is higher than global prevalence, affecting more than half of the Thai population ^5^.

Rome IV criteria for diagnosis classifies functional dyspepsia (FD) into two groups based on symptoms; (1) postprandial distress syndrome (PDS), consisting of postprandial fullness and early satiety, and (2) epigastric pain syndrome (EPS) ^6^. Currently, proton pump inhibitors (PPI) are regarded as an effective treatment for FD but ineffective in relieving PDS symptoms ^6^. Therefore, physicians frequently consider prescribing other agents for these patients.

 Pathogenesis of FD is likely complex and multifactorial. The factors that cause PDS comprise delayed gastric emptying time, impaired gastric accommodation, and gut inflammation ^6^. In addition, psychosocial factors such as anxiety, depression and psychiatric disorders also induce pathogenesis ^6^.

 Simethicone is a defoaming agent. Foam, formed by gas in the gastrointestinal tract (GI) and gastric mucous, is a cause of fullness if it accumulates in GI tract ^7^. Therefore, foam reduction can increase gastric emptying time and relieve postprandial fullness ^8– 13^. Many studies have found that simethicone has efficacy for treatment of dyspepsia and no serious adverse reaction ^14– 17^.

*Curcuma longa* is a Thai herb that effectively relieves flatulence ^18^. Previous rodent studies ^19– 21^ documented that *Curcuma longa* can decrease gut inflammation via its active ingredient curcumin (R = OCH _3_, R' = OCH _3_). Curcumin inhibits many proinflammatory enzymes such as cyclooxygenase-2, 5-lipoxygenase, and inducible nitric oxide synthase enzymes etc. Not only does it have an anti-inflammatory effect, but curcumin also increases gastric emptying time and reduces depressive symptoms via the brain-gut axis ^22^. Thus, *Curcuma longa* is commonly used for FD treatment.

 Many human studies supported the efficacy of *Curcuma longa* compared with other agents for treatment of dyspepsia ^19, 22, 23^.A trial in 2007 found that taking a 2-g *Curcuma longa* capsule daily for four weeks indicated no significant difference with ranitidine for dyspepsia relief ^23^. A later study in 2016 showed that addition of curcumin on top of the standard anti-helicobacter regimen in patients with peptic ulcers was safe and improved symptoms of dyspepsia but did not enhance effect on the eradication of *Helicobacter pylori* infection ^24^.

 However, no current evidence of the efficacy of *Curcuma longa* as compared with simethicone for PDS symptoms. Thus, the aim of this present study was to assess the efficacy of *Curcuma longa* compared with simethicone in patients with PDS.

## Methods

### Patients

Adults (age 20–60 years) with FD, diagnosed during a routine clinical appointment by physicians working at any Social Medicine clinic of Khon Kaen Hospital (Khon Kaen, Thailand), on the basis of Rome IV criteria ^6^, were screened by nurse officers for participation then enrolled in the study by the principal investigator. Inclusion criteria included postprandial distress syndrome, no alarm features, and discontinuation of all GI drugs at least one week before randomization. Patients with a history of either simethicone or *Curcuma longa* allergy, gastric malignancy, gallstone or biliary obstruction, pregnancy, and on breastfeeding period were excluded. Written informed consent was obtained from all patients.

### Study design and oversight

The Khon Kaen Hospital Institute Review Board in human research approved the study protocol. This trial was registered with the Thai Clinical Trials Registry on 31 ^st^ January 2018; registration number, TCTR20180131001. This randomized, active-comparator, open-label trial was conducted at primary care clusters of Khon Kaen Hospital, and Nam Pong Community Hospital between July 2018 and February 2019. All the authors were involved in the design and performance of the study, which was conducted according to the Declaration of Helsinki. First research assistant (CT) used computer-generated simple randomization and sequentially labeled the number on opaque drug containers for concealment. After the principle investigator (NS) enrolled participants, the second research assistant (MJ) then assigned the concealed interventions in order. There was no deviation from the original trial protocol.

### Study treatment and procedures

Patients were randomly assigned to take 750 mg *Curcuma longa* capsule per day, 1500 mg of *Curcuma longa* capsule per day or 240 mg of simethicone per day for four weeks. All patients were educated on lifestyle modification, such as stopping drinking and smoking, decreasing spicy foods and the volume eaten per meal, and trying to choose foods with softer consistentcy. Female participants were given a urine pregnancy test, and were excluded if result indicated positive. Baseline characteristics were measured together with BMI and global overall symptom (GOS) scale (described below). Medication was administered orally 30 to 60 minutes after each meal (250 mg (one *Curcuma longa* capsule per meal), 250 mg (two *Curcuma longa* capsule per meal), or 80 mg (one simethicone tablet per meal) three times per day). Patient's visits were scheduled at the start of treatment and at the end of 2, 4, and 6 weeks (2 weeks after stopping treatment). All patients discontinued medication after week 4 and reported for recurrence of symptoms at week 6. Patients also completed daily logs of symptoms and adverse events during the week preceding each visit. At each visit, the patient, with the principle investigator (NS), completed the seven-point GOS scale for dyspepsia (which ranges from 1 to 7, with 1 indicating no problem, 2 indicating minimal problem (can be easily ignored without effort), 3 indicating mild problem (can be ignored with effort), 4 indicating moderate problem (cannot be ignored but does not influence my daily activities), 5 moderately severe problem (cannot be ignored and occasionally limits my daily activities), 6 indicating severe problem (cannot be ignored and often limits my concentration on daily activities), and 7 indicating very severe problem (cannot be ignored and markedly limits my daily activities and often requires rest) ^25^.

At the last visit, patients were asked to report their recurrence of symptoms and the date of recurrence after discontinuation of treatment.

### Endpoints

We focused on PDS symptoms, measured using the GOS scale, so we combined the early satiety score and postprandial score as the composite outcome. The two primary endpoints were the comparison of mean changes of composite outcome between groups from baseline to week 2 and week 4. Secondary end points were rates and durations of recurrences at week 6, and also adverse effectsas assessed by a daily log of adverse events. Post-hoc analyses included the changes from baseline in each group at week 2 and week 4.

### Statistical analysis

We calculated that a sample of 69 patients would provide adequate power for the proposed tests in this three-group study using the formula for sample size calculation to compare k means by one-way ANOVA pairwise, 2-sided equality ^26^. By substitution of mean in treatment group (µ _trt_) = -1.86 ^24^, mean in control group (µ _con_) = -1.30 ^24^, SD in each group = 0.65 ^24^, α = 0.05 and β = 0.20, r = 1, n = 22 per group was be derived.

SPSS version 24.0 was used. Mean changes from baseline to week 2 and week 4, in GOS scale, were analyzed with one-way ANOVA. Dichotomous endpoints (recurrent rates) were compared among the groups with the use of Chi-squared and Z-test. The duration of symptoms was compared using Kruskall-Wallis test. Means among the groups were also analyzed using one-way ANOVA. To compare means in each group (before and after), a paired t-test was used. Bonferroni post-hoc test was used for post-hoc analysis.

## Results

### Study participants

**Figure 1.**
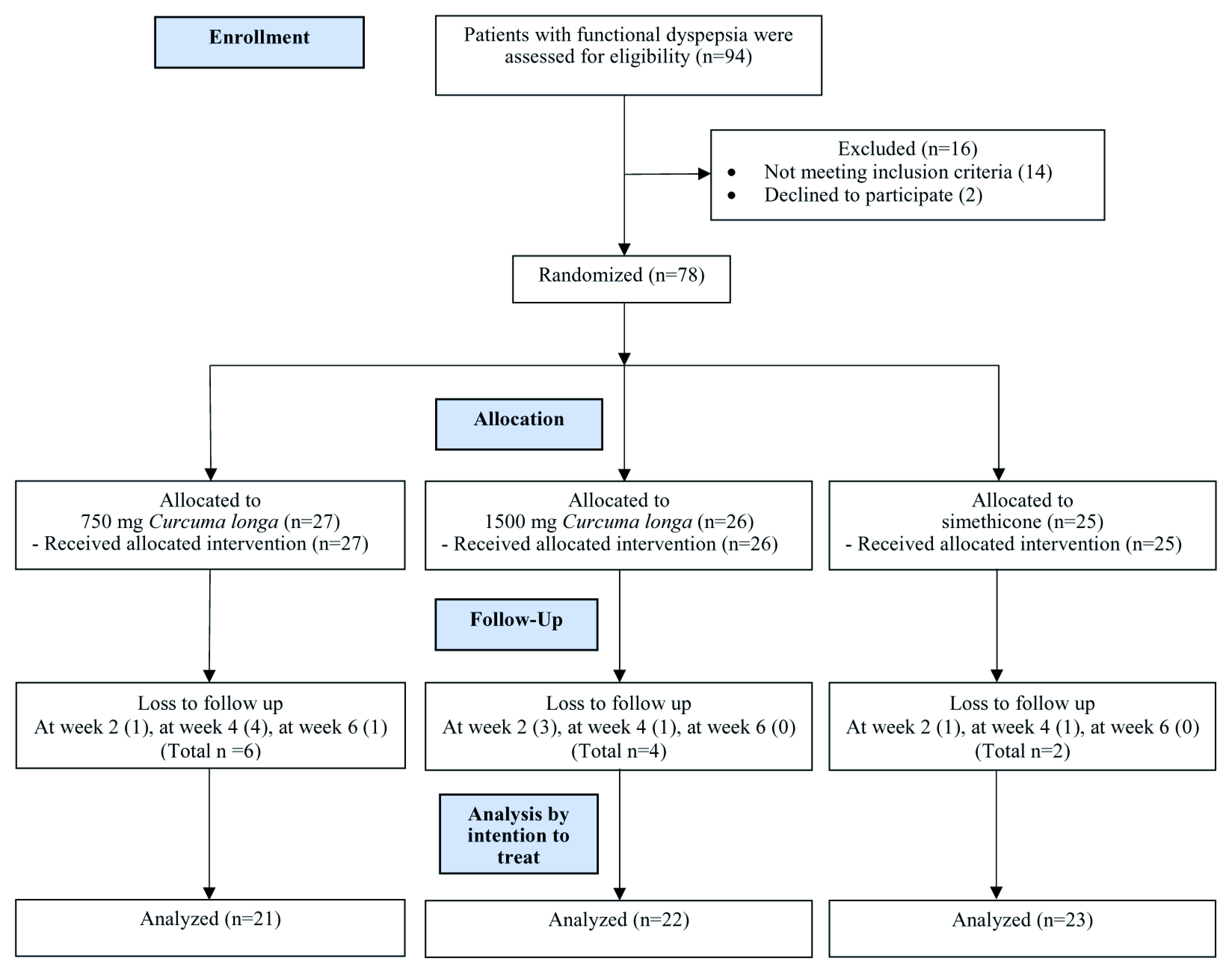
CONSORT diagram.

A total of 94 patients with functional dyspepsia were assessed for eligibility. There were 16 patients excluded from study due to not meeting inclusion criteria (n= 14) and declined to participate (n=2). A total of 78 patients underwent randomization. There were 27 in the 750 mg *Curcuma longa* group, 26 in 1500 mg *Curcuma longa* group and 25 in the simethicone group; there were 6, 4, and 2 patients lost to follow-up in each group, respectively. Intention-to-treat was used for data analysis, with n=21 in 750 mg *Curcuma longa*, n=22 in 1500 mg *Curcuma longa*, and n=23 in simethicone ( Figure 1). The characteristics of the patients at baseline were similar across study groups ( Table 1). However, in both *Curcuma longa* groups, patients were slightly overweight (BMI, 23.0-24.9 kg/m ^2^), while in the simethicone group, patients were normal weight (BMI, 18.5-22.9 kg/m ^2^). Participant characteristics, alongside all variables assessed, are available as *Underlying data* ^27, 28^.

**Table 1.** Baseline characteristics of the patients.

Characteristic	750 mg Curcuma longa N = 27	1500 mg Curcuma longa N = 26	Simethicone N = 25	P-value
**Female sex, n (%)**	19 (73.1)	20 (76.9)	18 (69.2)	0.856
Age, years *	38.0±13.8	41.4±12.9	36.2±12.0	0.348
Weight, kg *	60.4±11.7	63.3±10.3	57.5±10.4	0.166
Height, cm *	158.0±8.2	159.3±7.1	160.3±7.6	0.551
BMI, kg/m2 *	24.2±4.4	24.9±3.8	22.4±3.8	0.064
**Smoking, no (%)**				0.514
**Never**	25 (92.6)	22 (84.6)	20 (80.0)	
**Former**	2 (7.4)	2 (7.7)	3 (8.0)	
**Current**	0	2 (7.7)	4 (16.0)	
**Alcohol, n (%)**				0.169
**Never**	21 (77.8)	24 (92.3)	18 (72.0)	
**Former**	1 (3.7)	2 (7.7)	2 (12.0)	
**Current**	5 (18.5)	0	4 (16.0)	
**EGD approved FD, n (%)**	2 (7.4)	2 (7.7)	4 (16.0)	0.517
Duration of symptoms, years **	3.0 (5.5)	2 (4.0)	2 (4.0)	0.522
**Previous treatment, n (%)**	23 (85.2)	24 (92.3)	23 (92.0)	0.627
Global overall symptom scale *				
** Epigastric pain**	4.2±1.5	3.5±1.6	4.0±1.9	0.283
** Heartburn**	2.4±1.5	2.3±1.5	2.8±1.9	0.492
** Upper abdominal bloating**	3.8±1.7	3.8±7.8	3.7±2.0	0.967
** Excessive belching**	3.2±1.8	2.5±1.7	2.8±1.7	0.369
** Nausea**	3.0±1.7	2.0±1.4	2.3±1.6	0.777
** Early satiety**	4.4±1.7	4.4±1.7	4.4±1.7	0.988
** Posprandial fullness**	5.6±1.0	5.5±1.3	5.5±1.2	0.906

*Plus minus values are means ± SDs. **Values are medians (IQRs).

### Primary outcomes

After 2 weeks, there was no significant difference in mean change of PDS symptoms among three groups [-4.1 (-4.5, -2.6) vs -4.3 (-5.2, -3.3) vs -4.2 (-4.8, -3.5), P=0.954]. Over a period of 4 weeks, patients who received simethicone, as compared with those who received *Curcuma longa*, had a greater reduction (improvement) in the composite outcomes of PDS symptoms, but there was no statistically significant difference [-4.6 (-5.7, -3.6) vs -5.4 (-6.6, -4.1) vs -6.2 (-7.2, -5.2), P=0.122] ( Table 2).

**Table 2.** Comparison of before-after treatment GOS scale at the end of 2 and 4 weeks.

Global Overall Symptom (GOS) scale of dyspepsia	Mean changes of GOS between 2 and 0 week			Mean changes of GOS between 4 and 0 week		
	750 mg Curcuma longa n = 26	1500 mg Curcuma longa n = 23	Simethicone n=24	750 mg Curcuma longa n = 22	1500 mg Curcuma longa n = 22	Simethicone n=23
	Mean (95%CI)	Mean (95%CI)	Mean (95%CI)	Mean (95%CI)	Mean (95%CI)	Mean (95%CI)
**Epigastric pain**	-1.7 (-2.2, -1.1) P<0.001	-1.5 (-2.1, -0.8) P<0.001	-1.5 (-2.1, -1.0) P<0.001	-2.0 (-2.7, -1.3) P<0.001	-2.0 (-2.8, -1.3) P<0.001	-2.3 (-3.1, -1.5) P<0.001
**Heartburn**	-0.7 (-0.8, -0.2) P=0.016	-0.9 (-1.7, -0.2) P=0.012	-1.0 (-1.6, -0.5) P=0.001	-0.5 (-1.0, 0.0) P=0.053	-0.9 (-1.7, -0.8) P=0.034	-1.5 (-2.3, -0.7) P=0.001
**Upper abdominal bloating**	-1.5 (-2.1, -0.6) P<0.001	-1.7 (-2.4, -1.0) P<0.001	-1.6 (-2.3, -0.8) P<0.001	-1.9 (-2.9, -0.9) P=0.001	-1.9 (-2.8, -0.9) P=0.001	-2.3 (-3.2, -1.4) P<0.001
**Excessive belching**	-0.7 (-1.2, -0.1) P=0.042	-0.7 (-1.4, 0.2) P=0.096	-0.8 (-1.5, -0.2) P=0.026	-1.2 (-2.1, -0.3) P=0.010	-0.8 (-1.8, 0.2) P=0.107	-1.4 (-2.2, -0.6) P=0.002
**Nausea**	-0.5 (-1.3, 0.2) P=0.100	-0.3 (-0.3, 0.1) P=0.162	-0.4 (-1.0, 0.3) P=0.214	-1.0 (-1.9, -0.1) P=0.026	-0.2 (-0.8, 0.3) P=0.381	-0.7 (-1.5, 0.0) P=0.064
**Early satiety**	-1.7 (-2.1, -0.8) P<0.001	-1.6 (-2.2, -1.1) P<0.001	-1.7 (-2.2, -1.1) P<0.001	-1.6 (-2.4, -0.9) P<0.001	-2.2 (-2.9, -1.5) P<0.001	-2.8 (-3.4, -2.1) P<0.001
**Postpandrial fullness**	-2.4 (-2.6, -1.7) P<0.001	-2.6 (-3.1, -2.1) P<0.001	-2.5 (-2.9, -2.0) P<0.001	-3.0 (-3.6, -2.4) P<0.001	-3.1 (-3.8, -2.5) P<0.001	-3.4 (-3.9, -2.8) P<0.001
**Plus score of PDS (Early satiety** **and Postpandrial fullness)**	-4.1 (-4.5, -2.6) P<0.001	-4.3 (-5.2, -3.3) P<0.001	-4.2 (-4.8, -3.5) P<0.001	-4.6 (-5.7, -3.6) P<0.001	-5.4 (-6.6, -4.1) P<0.001	-6.2 (-7.2, -5.2) P<0.001

**Figure 2.**
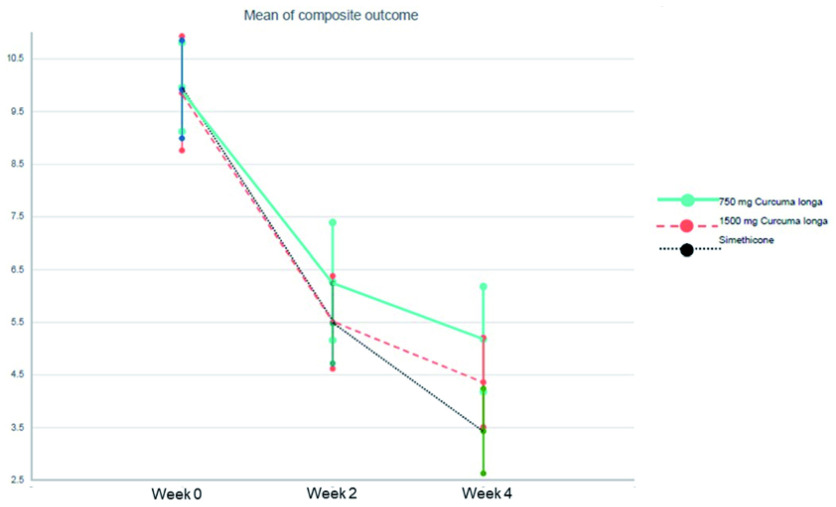
Mean differences of Global Overall Symptom score between three groups at the end of 2 and 4 weeks.

Figure 2 shows the mean differences in GOS between three groups at the end of 2 and 4 weeks. When calculating mean differences of treatment effect between *Curcuma longa* groups and simethicone, there was no significant difference of treatment effect among two pair-wise comparisons (group 3 vs group 1 and group 3 vs group 2) at weeks 2 and 4 ( Table 3).

**Table 3.** Comparison of treatment effect by composite outcomes.

Comparison of treatment effect between groups at week 2		P-value
**Treatment effect: mean differences in change (95% CI)**		0.954 ^a^
**Simethicone vs 750 mg** *Curcuma longa*	-0.09 (-1.54, 1.36)	> 0.999 ^b^
**Simethicone vs 1500 mg** *Curcuma longa*	0.09 (-1.40, 1.58)	> 0.999 ^b^
**Comparison of treatment effect between groups at week 4**		**P-value**
**Treatment effect: mean differences in change (95% CI)**		0.122 ^a^
**Simethicone vs 750 mg** *Curcuma longa*	-1.54 (-3.35, 0.28)	0.123 ^b^
**Simethicone vs 1500 mg** *Curcuma longa*	-0.81 (-2.62, 1.00)	0.828 ^b^

^a^By one-way ANOVA. ^b^Pairwise comparison of mean differences by Bonferroni post-hoc test.

### Secondary outcomes

***Comparison of before and after treatment using the GOS scale at the end of 2 weeks***. In Table 2, the 750 mg *Curcuma longa* group showed a significant reduction in all items of GOS scale except nausea which similar in simethicone group. However, in the 1500 mg *Curcuma longa* group indicated a significant reduction in almost all items of GOS scale except excessive belching and nausea.

***Comparison of before and after treatment using the GOS scale at the end of 4 weeks***. Over a period of 4 weeks, the participants among three groups showed significant improvement of their symptoms unless heartburn in 750 mg *Curcuma longa*, excessive belching and nausea in 1500 mg *Curcuma longa*, and nausea in simethicone ( Table 2).

***Rate of recurrence***. After discontinuing treatment for 2 weeks (washout period), The patients with 1500 mg *Curcuma longa* reported the highest rate of recurrence, 45.5%, followed by the patients with 750 mg *Curcuma longa*, 42.9% and the lowest rate was in simethicone group, 13.6% ( Table 4). In addition, the rate of symptom recurrence was found statistically significant among three groups (P=0.047).

**Table 4.** Rate and duration of recurrences.

Rate or duration	750 mg Curcuma longa (N=21)	1500 mg Curcuma longa (N=22)	240 mg Simethicone (N=22)	P-value
**Recurrence patient -no. (%, 95%CI)**	9 (29.3%, 95%CI 3.7-54.8%) *	10 (31.9%, 95%CI 6.5-7.1%) *	3	0.047 ^a^ 0.032 ^b^ 0.020 ^b^
**Mean duration of recurrence - day,** **mean (95%CI)**	4.1 (1.0, 7.2) **	4.5 (1.0, 8.0) **	4.2 (-6.1, 14.6) **	0.984 ^a^

*Proportion differences when compared to simethicone with its 95%CI (by Z-test) **The day after day 28 ^th^ ^a^One-way ANOVA ^b^Chi-square

***Duration of recurrence***. There was no significant difference in the duration of recurrence between groups ( Table 4).

***Adverse events***. There was no any patient who needed to discontinue treatment due to the serious adverse events. Non-serious adverse events were reported in 8 cases (11.9%) from the patients who receive *Curcuma longa*, including nausea, diarrhea, fever, dizziness and headache ( Table 5).

**Table 5.** Adverse events.

Adverse events	750 mg Curcuma longa, n (N=22)	1500 mg Curcuma longa, n (N=22)	240 mg Simethicone, n (N=23)
**Nausea**	1	1	1
**Fever**	2	0	0
**Diarrhea**	1	0	0
**Others**	1	0	1

## Discussion

The main limitation of this study is that it is an open-label trial, in which blinding was not performed. There was no co-intervention, but few attrition biases. Although this is an open-label trial, we performed the allocation concealment and a good randomization that the results of similar characteristics among three treatment groups. Due to validity of the outcomes measured with precise 95% CIs in Table 3, our findings are summarizable and generalizable to all similar settings and populations.

 The efficacy of *Curcuma longa* showed non-inferiority to simethicone according to the composite outcome of PDS symptoms among three treatment groups had no significant difference at week 2 and week 4. Our findings were similar to the findings of Sirijarugul and Pongchaidecha ^23^ and Khonche *et al* ^24^.

The study data also provide evidence of four important aspects of dyspepsia treatment. First, from baseline characteristics, there were more female participants. This is similar to the most recent meta-analysis in 2014 ^1^. Data from this prior study indicated a greater prevalence of dyspepsia in the women from 312,415 samples (OR 1.24; 95% CI 1.13 to 1.36) ^1^. The other characteristics were also accordant with previous studies ^23, 24^.

 Second, our findings showed that *Curcuma longa* groups were also effective in different doses. Suprisingly, the 1500 mg group developed a higher symptom recurrence rate. Therefore, 750 mg *Curcuma longa* per day should be the recommended dose for FD.

 Third, the simethicone group developed significant lower rate of symptom recurrence. To explain this phenomenon, these patients were normal weight from obesity Asian criteria (BMI, 18.5-22.9 kg/m ^2^) ^29^. On the other hand, in both *Curcuma longa* groups, patients were slightly overweight (BMI 23.0-24.9 kg/m ^2^) that associated with the greater prevalence of GI symptoms ^30, 31^.

Finally, the three treatment groups were safe for all participants, similar to previous studies ^19, 22– 24^, indicating that *Curcuma longa* can be used generally.

 Strengths of this study were; this was a randomized controlled trial that had a high quality of evidence, we studied a washout period, and we are the first who compare the efficacy of *Curcuma longa* and simethicone. On the other hand, our limitations were; having lower sample size than calculated due to loss to follow up patients that might have less power of study and dyspepsia associated with the multifactorial factor such as environment, various types of food, and participant behaviors. Despite we randomly assigned the treatments, it could not eliminate all confounders. So the outcomes could be imprecise.

## Conclusion

In conclusion, *Curcuma longa* had significant effects on reduction of FD, similar to simethicone after 2 and 4 weeks, but the recurrence rate (i.e. the proportion of reappearance) of dyspeptic symptoms was slightly significantly higher without serious adverse events.

## Data availability

### Underlying data

Figshare: CurcumaUnderlyingData. https://doi.org/10.6084/m9.figshare.9962723.v1 ^27^.

This project contains all de-identified variables assessed in this study.

Figshare: CurcumaDataDictionary. https://doi.org/10.6084/m9.figshare.10000625 ^28^.

This project contains the data dictionary for the underlying data, described above.

### Reporting guidelines

Figshare: CONSORT checklist for ‘Efficacy of Curcuma longa in treatment of postprandial distress syndrome: An open-label randomized-controlled trial’. https://doi.org/10.6084/m9.figshare.9962723.v1 ^27^.

Data are available under the terms of the Creative Commons Attribution 4.0 International license (CC-BY 4.0).
