# Supplementary material for: Efficacy of Curcuma longa in treatment of postprandial distress syndrome: An open-label randomized-controlled trial
Source: F1000Res. 2019 Oct 30;8:1827. [Version 1] doi: 10.12688/f1000research.20662.1 (PMC7809885; doi:10.12688/f1000research.20662.1)
Supplement: Peer review history

# Peer review: Reviewer response for version 1

**DOI:** 10.5256/f1000research.22724.r74786

**Published:** 2021-01-14

**Authors:** Somsook Santibenchakul

**Affiliations:** 1 Department of Obstetrics and Gynaecology, Faculty of Medicine, Chulalongkorn University, Bangkok, Thailand

Very nice work!

 Just one issue: I was curious about the following sentence.

 The patients with 1500 mg *Curcuma longa* reported the highest rate of recurrence, 45.5%, followed by the patients with 750 mg *Curcuma longa*, 42.9% and the lowest rate was in simethicone group, 13.6% (Table 4). 

 The percentages stated here were different from those in table 4. Could you please explain?

Is the work clearly and accurately presented and does it cite the current literature?

Yes

If applicable, is the statistical analysis and its interpretation appropriate?

Yes

Are all the source data underlying the results available to ensure full reproducibility?

Yes

Is the study design appropriate and is the work technically sound?

Yes

Are the conclusions drawn adequately supported by the results?

Yes

Are sufficient details of methods and analysis provided to allow replication by others?

Yes

Reviewer Expertise:

Epidemiologist

I confirm that I have read this submission and believe that I have an appropriate level of expertise to confirm that it is of an acceptable scientific standard.

# Peer review: Reviewer response for version 1

**DOI:** 10.5256/f1000research.22724.r62820

**Published:** 2020-05-12

**Authors:** Jie-ying Bai

**Affiliations:** 1 Laboratory Animal Center, Academy of Military Medical Sciences, Beijing, China; 2 Institute of Molecular Medicine, Peking University, Beijing, China

In this article, the effection of Curcuma Longa vs simeticone to postprandial distress was described. Two dose, 750mg/day and 1500mg/day, of C. longa were employed, the dose of simeticone was 240mg/day. Totally, the study design is appropriate, and enough literature was cited. According the data, we can find the similar effect between C. longa and simethicone. While, simethicone displayed less recurrence in this investment, which maybe show the tranditional Thai medicine, C. longa, is not a good alternative in treatment of postprandial distress syndrome.

 In addition, there are so many people suffered from dyspepsia in Thai according introduction of this article. There were only 94 patients with functional dyspepsia were assessed for eligibility in this open-label trial. The patient samples were deficiency. This study should employ an cohort research in Thailand.

Is the work clearly and accurately presented and does it cite the current literature?

Yes

If applicable, is the statistical analysis and its interpretation appropriate?

Yes

Are all the source data underlying the results available to ensure full reproducibility?

Yes

Is the study design appropriate and is the work technically sound?

Yes

Are the conclusions drawn adequately supported by the results?

Yes

Are sufficient details of methods and analysis provided to allow replication by others?

Yes

Reviewer Expertise:

Molecular epidemic, Immunogenetics

I confirm that I have read this submission and believe that I have an appropriate level of expertise to confirm that it is of an acceptable scientific standard.

# Response

**Published:** 2020-05-14

**Authors:** Bandit Chumworathayi

**Affiliations:** Khon Kaen University, Thailand

Thank you very much for your comments. Further research with larger sample size will be conducted.
